# Acute syphilitic chorioretinitis after a missed primary diagnosis: a case report

**DOI:** 10.1186/1752-1947-2-33

**Published:** 2008-02-01

**Authors:** Claudia Handtrack, Harald Knorr, Kerstin U Amann, Christoph Schoerner, Karl F Hilgers, Walter Geißdörfer

**Affiliations:** 1 Department of Nephrology and Hypertension, Krankenhausstraße 12, 91054 Erlangen, Germany; 2 Department of Ophthalmology, Schwabachanlage 6, 91054 Erlangen, Germany; 3 Institut of Pathology, Krankenhausstraße 8, 91054 Erlangen, Germany; 4 Institut of Clinical Microbiology, Immunology and Hygiene, Wasserturmstraße 3, 91054 Erlangen, Germany

## Abstract

**Introduction:**

Syphilis is well known as an infectious disease which can present with a large variety of symptoms. Clinical diagnosis can be difficult and may be complicated in modern medicine by immunosuppressive treatment and possible side effects of medication.

**Case presentation:**

We describe a rare case of placoid chorioretinitis due to *Treponema pallidum *which developed after the primary symptom of proteinuria was not recognized as a rare manifestation of syphilis. Diagnosis of syphilitic chorioretinitis and/or endophthalmitis was made by broad range amplification of the bacterial 16S ribosomal RNA gene obtained from vitreous after diagnostic vitrectomy.

**Conclusion:**

This case shows that clinicians should be alert in patients with proteinuria and chorioretinitis as they can represent rare manifestations of syphilis. Syphilis should be in the differential diagnosis of any unknown symptom and in the presumed side effects of medication.

## Introduction

Primary syphilis is characterized by a single cutaneous or mucosal lesion (hard chancre) at the site of infection, which is self-healing, but frequently followed by the secondary, disseminated stage with a variety of possible symptoms, including maculopapular rashes, mucous patches in the mouth, genital condylomata, alopecia, malaise with fever, and, in less than one percent of the cases, renal or ocular manifestations [1]. Laboratory diagnosis of syphilis by serological tests is well established, highly sensitive and specific.

Within the last decade a rising incidence of syphilis in the Western World has been reported, especially among men who have sex with men [2]. Today, the knowledge of even rare manifestations of the disease and the awareness of its increasing epidemiological relevance remain the key requirement for a correct and rapid diagnosis of *Treponema pallidum *infections by physicians. This problem is illustrated by our patient, who developed syphilitic chorioretinitis after the initial diagnosis of proteinuria was thought to result from focal segmented glomerulosclerosis (FSGS) and who was treated with immunosuppressive doses of prednisolone.

## Case presentation

A 35-year-old female patient with a medical history of myasthenia gravis, who was treated for more than 20 years with pyridostigmine, was admitted to our department of ophthalmology because of acute loss of vision. Three months prior to admission the patient suffered from bronchitis, accompanied by puffiness and ankle edema, presumably due to nephrotic range proteinuria, and arthralgias. Furthermore, she noted an intermittent exanthema on the flexor sides of both arms, on her calves and on the chest below her breasts. Her general practitioner found a nephrotic range proteinuria (9 g/d), subfebrile temperatures and a very high erythrocyte sedimentation rate of 22 mm per hour. Two weeks later she was referred to our nephrology department for an elective renal biopsy, which, unfortunately, did not yield a clear diagnosis upon immuno-histopathological examination. Mild mesangial hypercellularity and interstitial leukocyte infiltration were interpreted as being compatible with an early stage of primary focal segmented glomerulosclerosis (FSGS). The patient was treated with an angiotensin-converting enzyme inhibitor, a statin, and prednisolone (1 mg/kg bodyweight, i.e. 60 mg/d) and seen regularly for follow-up visits in the outpatients' clinic.

One month after the onset of therapy the patient started suffering from severe stomatitis with the detection of *Candida albicans*, which was locally treated with an amphotericin B suspension. At that time the proteinuria had improved and was 1 g/d. One week later the patient showed multiple oral aphthous lesions, from which herpes simplex virus (HSV) DNA could be amplified. The patient therefore was treated with oral aciclovir. She also reported hoarseness and loss of hair which were interpreted as side effects of the steroid therapy, and a maculopapulous exanthema of the palms which was diagnosed as a potential symptom of ACE inhibitor intolerability. Two weeks later the stomatitis had improved markedly, and the palmar exanthema had almost resolved.

**Figure 1:**
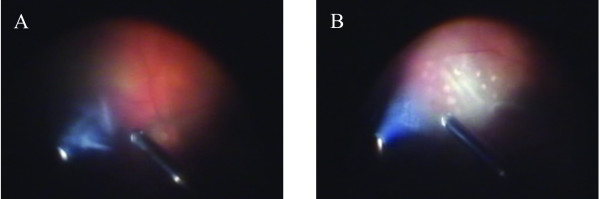
**Findings during vitrectomy**. (A) Whitish cloudy cords. (B) Retinitis with multiple ill-defined spots.

14 days prior to the present admission she had experienced several episodes of blurred vision. The sudden bilateral total loss of vision to hand movements led to an emergency admission to the university ophthalmology department. Ophthalmoscopy revealed whitish, cloudy veils in the vitreous body and bilateral retinitis with multiple white, ill-defined spots, particular in the immediate vicinity of large retinal vessels, highly suggestive of mycotic endophthalmitis (Fig. 1, movie in additional file 1). The patient was immediately treated with intravenous fluconazole and cefotiam, which did not lead to improvement of the retinal lesions. Four days later, a diagnostic vitrectomy of the left eye was performed.

Microscopically the vitreous fluid contained leukocytes, but no bacteria or fungi. Aerobic and anaerobic cultures remained sterile after 7 days, and PCR tests for viral DNA including cytomegalovirus were negative. A broad range PCR for bacterial 16S ribosomal RNA gene (rDNA) yielded a 805 bp fragment that showed 100 % identity to the 16S rDNA sequence of *T. pallidum *[GenBank: AE000520]. Diagnosis of syphilis was confirmed by serological tests (Table 1), penicillin G (5 × 10^6 ^units 5 times per day i.v.) was given for two weeks, and prednisolone was discontinued. During the following weeks and months the stomatitis, erythema and proteinuria resolved completely, and successful treatment was confirmed by a significant decline of *T. pallidum*-specific antibodies (Table 1). The patient's vision also improved to finally 0.63 for the right eye, but remained impaired in the left eye (0.07). Upon specific questioning the patient admitted a single episode of casual sexual intercourse 4 months prior to the acute loss of vision as the possible time of infection. A syphilitic chancre was not noticed by the patient.

**Table 1:** Serological assay results at primary diagnosis and follow-up

Assay	Time of serological diagnosis					
	
	Primary diagnosis	1 month	3.5 months	9.5 months	1 year 4 months	1 year 8 months
TPPA	1:10240	1:2560	1:1280	1:320	1:320	1:160
FTA-Abs	pos.	n.d.	n.d.	n.d.	n.d.	n.d.
IgM FTA-Abs	1:80	1:10	neg.	neg.	n.d.	n.d.
Cardiolipin CFR	1:320	1:160	1:80	1:20	1:5	1:5

CFR, Complement-fixation reaction; FTA-Abs, fluorescent treponemal antibody absorptions test; TPPA, *Treponema pallidum *particle agglutination assay; n.d., not determined; neg., negative; pos., positive.

## Discussion

In this case of a 35-year-old female patient, diagnosis of syphilis was only made after diagnostic vitrectomy. The initial symptom of our patient was a nephrotic range proteinuria of unknown origin. Kidney involvement in *Treponema pallidum *infection occurs in various forms including nephrotic syndrome [3], membranous [4], or minimal change glomerulonephritis [5], but is rare and not highly indicative of syphilis. Unfortunately, the majority of the other primary clinical symptoms, which, retrospectively, were most likely caused by the infection with *T. pallidum*, were either non-specific and/or could be interpreted as possible side effects of the concurrent medication: bronchitis, arthralgia, erythema, loss of hair. Moreover, there was laboratory evidence supporting the diagnosis of an intermittent viral infection (HSV). Only the severe complication of chorioretinitis that required hospital admission and the lack of response to antifungal and antibacterial treatment necessitated vitrectomy and further diagnostic tests. The cellular infiltration of the vitreous and the white retinal spots, combined with the patient's recent history of *C. albicans *stomatitis and immunosuppressive treatment, strongly suggested infectious, most probably mycotic, endophthalmitis. Finally, universal bacterial PCR led to the diagnosis of syphilitic placoid chorioretinitis. This method has proven a very useful diagnostic tool, because even non-culturable and non-viable bacteria after antibiotic treatment can be detected [6]. Detection of *Treponema pallidum *DNA by specific PCR in vitreous specimens has only recently been reported for the first time [7,8]. Diagnosis of syphilis by PCR is especially required and reasonable in select cases, when serological assays are of limited use or when the infection of a given organ system needs to be confirmed [9].

Manifestations of ocular syphilis are manifold and are predominantly found in HIV-positive or immunosuppressed patients [10], but it is not clear, if this just reflects the higher prevalence of syphilis within these groups, or if modification of the immune system really promotes infection [11,12]. Our patient was HIV-negative, but was under mild immunosuppression for supposed FSGS. In a previous study *Treponema pallidum *DNA was detected in a quarter of spinal fluid samples from patients independent of their HIV status, and more often in early syphilis (40 %) than in secondary (23 %) or early late stage (20 %). This indicates that invasion of the CNS is common in syphilis [13]. Considering its known hematogenous dissemination, it is not surprising that *Treponema pallidum *can also be found in vitreous. The detection of *Treponema pallidum *DNA in the vitreous of our patient and the rapid response to treatment with penicillin G strongly suggests that the fulminant intraocular pathology seen in our patient was directly related to the presence of viable *Treponema*. Likewise, the complete remission of proteinuria after penicillin treatment without further corticosteroid therapy combined with the observation that nephrotic-range proteinuria did not re-occur after more than two years, points towards initial syphilitic nephropathy.

## Conclusion

In conclusion, this case illustrates the difficulties in diagnosing syphilis in a situation, where the initial manifestation of infection was unusual (mild nephrotic syndrome), obvious sexual risk factors like prostitution were absent, and more typical symptoms of syphilis (e.g., palmar erythema, hair loss) could be interpreted as medication-induced complications. Considering the chameleonic behaviour of syphilis and its recent increase in the Western World [14], specific questioning and serological testing for syphilis should always be performed whenever the etiology of organ symptoms remains unclear.

## Competing interests

The author(s) declare that they have no competing interests.

## Authors' contributions

C Handtrack took care of the patient during hospitalization and drafted the manuscript. H Knorr performed ophthalmoscopy and vitrectomy. KU Amann carried out immunohistopathological examination of the renal biopsy. C Schoerner performed the microbiological diagnostics. KF Hilgers participated in the patient's care and in drafting the manuscript. W Geißdörfer performed the molecular diagnostics and helped to draft the manuscript. All authors read and approved the final manuscript.

## Consent

Written informed consent was obtained from the patient for publication of this Case report and any accompanying images. A copy of the written consent is available for review by the Editor-in-Chief of this journal.

## Supplementary Material

Additional file 1 Ophthalmoscopic findings during vitrectomy. The video shows the whitish cloudy cords and the white retinal spots found during vitrectomy. Click here for file
